# Value chain analysis of medicinal plants in Geoparks: Livelihoods of ethnic minorities based on non-timber forest products (NTFPs) in Vietnam

**DOI:** 10.1371/journal.pone.0324746

**Published:** 2025-07-23

**Authors:** An Thinh Nguyen, Kien Huy Ngo, Nam Xuan Ngo, Ngoc Anh Le, Ngoc Trinh Phuong, Do Thi Thao, Huyen Trang Le, Chi Linh Vu, Thi Toan Luu, Van Quy Khuc, Hao Thi Nguyen, Duc Bao Nguyen

**Affiliations:** 1 VNU University of Economics and Business, Vietnam National University, Hanoi, Vietnam; 2 National Institute of Agricultural Planning and Projection (NIAPP), Hanoi, Vietnam; 3 Vietnam Sanitary and Phytosanitary Notification Authority and Enquiry Point (SPS Vietnam), Hanoi, Vietnam; 4 Faculty of Business Administration, Ton Duc Thang University, Ho Chi Minh City, Vietnam; 5 Tan Trao University, Tuyen Quang, Vietnam; 6 School of Hospitality and Tourism, Hue University, Hue, Vietnam; 7 VNU School of Interdisciplinary Sciences and Arts (VNU-SIS), Hanoi, Vietnam; Lusofona University of Humanities and Technologies: Universidade Lusofona de Humanidades e Tecnologias, PORTUGAL

## Abstract

Non-timber forest products (NTFPs) support the livelihoods and economic activities of households and communities in both rural and urban areas worldwide. This study applies the value chain analysis (VCA) approach to Jiaogulan *(Gynostemma pentaphyllum)*, a local NTFP in the Cao Bang Geopark, Vietnam. A semi-structured questionnaire was used to survey 106 stakeholders involved in different stages of the Jiaogulan value chain, including agricultural experts (extension officers and farmer association representatives), harvesters, traders, primary processors, secondary processors, distributors, and consumers. The findings present a comprehensive mapping of the Jiaogulan value chain, illustrating the flow of the product from harvest to end consumer and quantifying the added value at each stage of the process. The study highlights the value captured by households involved in the primary market channels and assesses how this added value contributes to household income levels. The distribution of economic benefits across different actors within the value chain is also examined, identifying potential inefficiencies and disparities that need to be addressed to improve equity and economic outcomes for marginalized groups. Recommendations are made to strengthen production and cooperation among Jiaogulan value chain actors through technical training, infrastructure investment, e-commerce development, and policy support for international standards and medicinal value chains to enhance product quality, market access, and sustainable development in Cao Bang Geopark.

## 1. Introduction

Non-timber forest products (NTFPs) refer to resources that can be extracted from forest ecosystems for household use or commercial purposes. These resources hold social, cultural, and religious significance [1,2]. NTFPs provide food, energy, fibers, medicine, shelter, and cultural artifacts, serving as critical resources for some of the world’s most impoverished populations, as well as for a significant portion of those in relatively better living conditions [3–6]. In traditional forest communities, NTFPs play a crucial role in household survival strategies, supplying essential nutrients such as carbohydrates, fats, proteins, and micronutrients like minerals [7]. These products may serve as staple foods for those living near forests or as coping mechanisms when access to other agricultural resources is limited. Furthermore, NTFPs are used as livestock feed, highlighting their significance within the broader food supply chain.

Beyond subsistence, NTFPs provide nutrition, health care, and construction materials for households in both rural and urban areas, particularly in developing countries [8]. They also generate substantial economic value and create numerous employment opportunities, drawing global attention for their potential to enhance rural livelihoods [9,10]. NTFPs contribute significantly, ranging from a small percentage to over 50% of the total income of households [11–13]. They account for the highest proportion of income among poor households and underdeveloped communities, especially in mountainous and remote areas [8]. The commercialization of NTFPs at local, national, and regional levels tends to increase, providing cash income to households [14,15]. For instance, in southwestern Malawi, most households trade at least one type of NTFP, generating an annual income between 20 and 456 USD [16]. However, the sustainability of NTFP trading is challenged by the short value chain and the domination of traders and intermediaries [17].

Value chain analysis (VCA) provides a framework to examine the processes and actors involved in the production, distribution, and consumption of NTFPs, with the goal of identifying opportunities to increase value and improve the livelihoods of those involved. VCA has been applied to the commercialization of NTFPs in various regions worldwide [18], including the honey value chain in the Philippines [19], the agarwood value chain in Sudan [20], the palm fruit value chain in Brazil (De Sousa et al., 2018), and the spice value chain in Cameroon [21]. The value chain map of NTFPs describes the links among all the actors and the transactions from producers or harvesters to final consumers, showing the sequence of stages from harvesters, processors, and traders to final consumers [18,22]. VCA helps describe the market for NTFPs and assess the value chain’s performance, while providing information on the market share and price distribution that each actor secures [1,23,24]. This analysis is useful for identifying barriers that prevent poor people from fully benefiting from NTFP production activities.

Although most NTFP markets require limited input capital, especially in local markets, profits from trading NTFPs tend to be low. Higher trading volumes could yield better incomes but require more investment in storage and transportation [24]. Study findings from VCA suggest effective solutions for improving the equitable distribution of earnings from NTFPs [25,26] (Mitchell et al., 2011; Wynberg et al., 2014). VCA of NTFPs has been used to enhance local economic development, household livelihoods, community development, and poverty reduction in mountainous and remote areas [27–29].

Adapting to changes in agricultural commodities and improving smallholder farmer participation in NTFP value chains is emerging as a strategy for poverty alleviation [29]. Developing NTFP value chains not only enhances smallholder farmers’ access to markets, inputs, and credit but also boosts production efficiency [30]. In Vietnam, the government has increasingly prioritized the development of agricultural value chains [31]. With a target of reaching $20 billion in exports for timber and NTFPs by 2025, authorities at various levels are focusing on enhancing all aspects of NTFP value chains. This includes efforts in production, conservation, land-use planning, cultivation, harvesting, and processing, particularly in ethnic minority and highland regions.

Over the past few years, Vietnam has implemented an agricultural restructuring project aimed at promoting sustainable rural development and improving agricultural productivity. By 2021, 56 of 63 provinces and cities had issued policies to encourage linkages in the production and consumption of agricultural products. A total of 4,028 agricultural cooperatives were established, working with 1,867 enterprises to promote the production, harvest, processing, and consumption of agricultural products. As an important type of agricultural product in mountainous rural areas, NTFPs sustain the livelihoods of local households and communities [32].

In the northern mountains of Vietnam, Jiaogulan *(Gynostemma pentaphyllum)* (‘Giảo cổ lam’ in Vietnamese) stands out as one of the most notable NTFPs. It grows under the forest canopy in the limestone mountains of northern Vietnam, including Hoa Binh, Lao Cai, and Cao Bang provinces. Jiaogulan is used as a medicinal herb known for its health benefits [32]. Over the past two decades, scholars have explored its medicinal potential, leading to the isolation of over 200 compounds with diverse therapeutic effects, such as anticancer, anti-obesity, anti-inflammatory, and antioxidant properties [33]. Several conditions, including diabetes, hepatitis, and cardiovascular diseases, are treated in traditional Chinese medicine using Jiaogulan [34].

In Cao Bang, a border province between Vietnam and China, the local Tay community has long processed Jiaogulan as dried tea. It is estimated that Cao Bang produces 5–7 tons of Jiaogulan annually [35]. Jiaogulan is sold in various forms, including as raw material for food and medicinal products, as well as dried tea. However, overexploitation of natural Jiaogulan has led to its scarcity, and it is now listed as an endangered plant in the Vietnam Red Data Book (2007). The Jiaogulan value chain supports local economic development, contributes to hunger eradication, and alleviates poverty in remote areas.

The objectives of this study are to apply VCA to Jiaogulan in Cao Bang Geopark to better understand the contribution of this plant to ethnic minority communities in the locality. Mapping the value chain identifies the actors involved in the production, processing, and distribution of Jiaogulan, clarifying how the product moves through various stages of the supply chain. Quantifying the economic value of Jiaogulan highlights their potential to increase household income and improve local livelihoods. Furthermore, the study assesses the impact of this value chain on poverty alleviation, revealing its potential for sustainable economic development in remote and rural areas of Vietnam. This not only benefits local communities but also opens opportunities for developing higher-value products from natural resources, contributing to the sustainable agriculture at both local and national levels in Vietnam.

## 2. Methodology

### 2.1. Study area

The study is conducted in the Cao Bang Geopark (CBGP), located entirely within Cao Bang province in the mountainous region of northern Vietnam. The park covers the districts of Ha Quang, Trung Khanh, Ha Lang, Quang Hoa, and parts of Hoa An, Nguyen Binh, and Thach An. CBGP spans more than 3,390 square killometers and was recognized as a global geopark by UNESCO in 2018. The park has a total area of 6,724.6 square killometers, with an average elevation of over 200 meters, and the border areas range from 600 to 1,300 meters in altitude. Sloping land accounts for more than 90% of CBGP’s total area. The geopark shares a 333 km border with China, with major border crossings including Ta Lung, Tra Linh, Soc Giang (Vietnam) and Thuy Khau, Long Bang, Binh Mang (China). The recognized values of CBGP include its rich geological history, spanning approximately 500 million years. The park contains marine sediments, fossils, minerals, and volcanic rocks. The limestone landscapes are home to 214 cultural and historical sites, as well as more than 130 unique geological heritage sites typical of tropical karsts, such as Ban Gioc waterfall, Nguom Ngao cave (Trung Khanh district), Ho Thang Hen lake (Tra Linh district), and Phja Oac-Phja Den National Park (Nguyen Binh district).

CBGP is home to around 250,000 people from nine ethnic minorities, including the Tay, Nung, and Dzao. Two intangible cultural heritages of these ethnic groups are recognized at the national level: the Then rituals of the Tay ethnic community and the Nang Hai Festival (Phuc Hoa district). Local culinary specialties associated with agricultural products in the region include Phja Den vermicelli (Nguyen Binh district), pears and black jelly (Thach An district), chestnuts and white jelly (Trung Khanh district), and Khau Sli Na Giang cake (Ha Quang district), along with dishes like Banh Cuon, Pho Chua, Coong Phu cake, Khao cake, roasted duck, Jiaogulan tea, and red Polygonum tea. CBGP is also rich in biodiversity, featuring 940 medicinal species across 585 genera and 156 families, many of which have high economic value. These species include Dang ginseng, Cat ginseng, Hoang Tinh, Ba Kich, Ramulus Ampelopsis tea, bitter tea, and honeysuckle. Particularly noteworthy is the indigenous population of Jiaogulan, with relatively large reserves growing wild on the limestone mountains of CBGP [32].

### 2.2. Data collection

The study employs the VCA framework proposed by Kaplinsky et al. (2000) [23] to examine the Jiaogulan value chain in the Cao Bang Geopark: mapping, describing, quantifying, and assessing economic impacts of the Jiaogulan value chain. Mapping the value chain involves delineating the sequence of production processes, business activities, and transactions among the actors engaged in the Jiaogulan value chain. The analysis identifies the roles of key stakeholders such as producers, processors, and distributors, as well as the roles of external supporters such as regulatory bodies, financial institutions, and technical service providers. It also examines the linkages between these actors, highlighting the formal and informal networks that facilitate the movement of Jiaogulan from harvest to consumption, including forward and backward linkages. Following the value chain mapping, the study quantifies the performance of the value chain in terms of production volume, market segmentation, and stakeholder roles. This includes analyzing the physical flow of Jiaogulan products across various nodes in the chain, measuring production capacity, and capturing data on market penetration across local, regional, and national segments. The rate of volume transfer from one actor to the next in the value chain across the chain is quantified. Also, the study assesses the economic impact by calculating the changes of parameter’s value at different stages of the Jiaogulan value chain, such as production, primary processing, secondary processing, and distribution. The analysis considers the potential for economies of scale and scope by examining factors such as prices, intermediary costs, value added, additional costs, net value added, and the proportion of net value added at each stage of the value chain.

In this study, value addition is as estimated based on stakeholder interviews, cost breakdowns provided by processors and traders, and market price differentials at various transaction points. To enhance transparency, we elaborate on the data collection process, including how production costs, labor inputs, processing expenditures, and retail markups are recorded. This approach provides an indicative assessment of value distribution among different actors, while we acknowledge that more rigorous economic modeling, such as cost-benefit analysis or input-output modeling, could further strengthen the findings.

Primary data for this study is gathered through direct interviews with stakeholders involved in the Jiaogulan value chain. To ensure data representativeness, the method of conditional quota sampling is employed, aligning with the value chain approach to collect detailed information from stakeholders. This approach allows for accurate representation of the heterogeneous groups involved in the value chain, and ensures that the socioeconomic and demographic variations within these groups are adequately captured. The interview process is structured according to a seven-step methodology proposed by Kumar (2005) [36]:

- Identifying interview issues (Step 1): This step involves defining the key issues and objectives to be addressed during the interviews, ensuring alignment with the research questions related to value chain mapping and economic impact assessment.
- Planning and design (Step 2): A comprehensive interview guide is developed, focusing on key variables such as production capacity, cost structures, market linkages, and socioeconomic impacts of Jiaogulan production and trade. An in-depth interview questionnaire was developed for the purpose of semi-structured interviews. The total of 106 stakeholders are selected to survey including 6 experts, 12 harvesters, 15 wholesalers, 16 pre-processors, 15 domestic processors, 12 retailers, and 30 consumers.
- Conducting interviews (Step 3): Interviews are carried out using a structured guide to maintain consistency in data collection across all stakeholder groups. Both email and face-to-face interviews are employed for expert and consumer respondents, while only face-to-face interviews are conducted for other respondent groups.
- Data documentation (Step 4): Detailed notes and interview transcripts are prepared, ensuring that all qualitative and quantitative data are accurately recorded for subsequent analysis.
- Data analysis (Step 5): The collected data is analyzed using descriptive statistical methods, financial indicators (such as gross margin, value-added ratios, and cost-to-benefit analysis), and value chain mapping tools. These analyses facilitate a comprehensive understanding of the Jiaogulan value chain, elucidating the economic linkages and profit distribution across different stages and actors in the chain. The findings contribute to a deeper understanding of the economic and social dynamics driving the Jiaogulan value chain in Cao Bang province, with broader implications for rural development and poverty alleviation strategies in mountainous regions of Vietnam.
- Verification (Step 6): Cross-validation methods are used to ensure the reliability and accuracy of the data, including triangulation with secondary sources and a comparison with historical data.
- Reporting (Step 7): The findings are compiled into a report, with results communicated based on pre-defined scoring criteria for evaluating the value chain’s economic performance and social impact.

The survey period coincided with the peak Jiaogulan harvesting season, running from March to May 2022. Table 1 outlines the respondent groups and information collected from each.

**Table 1:** Survey sampling and data collection from stakeholders.

No.	Interviewed actors	Collected information	Quantity of respondents	Interview methods
1	Experts (agricultural extension officers, farmers’ associations)	Experts offer in-depth insights into various aspects of the Jiaogulan value chain model, including production processes, market dynamics, supply chain management, and value addition practices. They are asked to share personal information relevant to their roles, such as their professional background, years of experience in the field, specific areas of expertise, and their involvement in Jiaogulan-related research, policy-making, or industry development. This information helps contextualize their perspectives and contributions to the value chain analysis.	6	Email and face-to-face interviews
2	Harvesters	Harvesters include farmers who harvest the herbs, local collectors, and outside collectors are asked to provide detailed information on their livelihood activities related to Jiaogulan cultivation, including the methods and techniques they use, the scale and frequency of harvesting, and the challenges they face. In addition, they share personal socioeconomic information such as household income, education level, family size, and any other sources of income, offering a comprehensive understanding of their living conditions and economic dependence on Jiaogulan cultivation.	12	Face-to-face interviews
3	Wholesalers	Wholesalers provide detailed information on their income sources, including earnings from Jiaogulan trading and other revenue streams. They describe trading dynamics, such as pricing strategies, seasonal demand-supply fluctuations, and negotiation practices. Additionally, they outline input-output relationships, covering raw material costs, availability, and their impact on profitability. Logistics details include transportation, storage, delivery schedules, and challenges in moving products from harvesters to markets. This data offers a comprehensive view of their role and challenges within the Jiaogulan value chain.	15	Face-to-face interviews
4	Pre-processors	Pre-processors provide detailed reports on their family-run facilities, including operational scale, family involvement, and processing techniques. They describe output linkages with value chain stakeholders and outline preliminary processing methods, such as washing, drying, sorting, and packaging. Additionally, they specify quality appraisal standards, detailing criteria like leaf size, color, and moisture content. This information highlights their role in maintaining Jiaogulan’s quality and consistency within the value chain.	16	Face-to-face interviews
5	Domestic processors	Domestic processors provide data on Jiaogulan volumes, detailing raw material intake and final output. They describe processing methods, such as drying, extraction, and powdering, based on market requirements. Additionally, they outline quality standards, including purity, active ingredient content, moisture levels, and packaging. This information clarifies the scale and quality control within the Jiaogulan processing segment.	15	Face-to-face interviews
6	Retailers	Retailers provide data on distribution channels, including domestic and international networks, partnerships, and online platforms. They outline quality evaluation criteria, such as packaging integrity, certification, and regulatory compliance. Additionally, they detail vendor contracts covering pricing, delivery schedules, order quantities, and payment terms. This information clarifies Jiaogulan’s movement through the supply chain and the business relationships facilitating it.	12	Face-to-face interviews
7	Consumers	Consumers provide insights into their Jiaogulan usage, including preferred forms (tea, capsules, extracts), consumption frequency, and purpose (health, medical, wellness). They indicate their familiarity with its benefits, side effects, and information sources. Additionally, they describe purchasing habits, including preferred outlets and brand preferences, as well as their sensitivity to price changes and response to promotions. This information offers a comprehensive view of consumer behavior and preferences.	30	Email and face-to-face interviews
	Total		106	

Table 1 shows that survey sampling and data collection from stakeholders. An in-depth interview questionnaire is developed to facilitate semi-structured interviews, ensuring a balance between guided inquiries and open-ended discussions. A total of 106 stakeholders are selected for the survey, comprising various key actors across different stages of the Jiaogulan value chain. The sample includes 6 experts with extensive knowledge of Jiaogulan cultivation, processing, and market trends; 12 harvesters engaged in different farming practices, representing both small-scale and larger agricultural operations; 15 wholesalers operating at regional and national levels; 16 preliminary processors involved in initial handling and preservation; 15 domestic processors who add further value to the product before it reaches consumers; 12 retailers selling Jiaogulan through different distribution channels; and 30 consumers from diverse demographic and geographic backgrounds.

To enhance the representativeness of the sample, participants are selected from various rural and urban contexts to capture the diversity of experiences, practices, and market conditions. The selection process considered key factors such as geographic distribution, scale of operations, and supply chain roles to ensure that perspectives from both traditional and commercialized sectors of the value chain were reflected. While the sample size of 106 stakeholders may not encompass all potential variations, our efforts are made to include a broad spectrum of participants, enabling meaningful insights into the dynamics of the Jiaogulan value chain in the studied Cao Bang geopark.

We acknowledge the potential variability in the depth and quality of responses due to the semi-structured interview method. To mitigate this, we ensure consistency by using a well-defined interview guide, allowing us to explore key themes while maintaining a level of comparability across responses. Additionally, interviewers are trained to probe deeper when necessary, ensuring that critical aspects are adequately covered.

Focus Group Discussions (FDGs) are incorporated as part of the study methodology to enhance the robustness of findings. Three FDGs are organized with the support of Cao Bang Geopark Management Board. These discussions allow for dynamic exchanges among stakeholders, enabling the identification of common themes, points of divergence, and collective perspectives on key issues within the Jiaogulan value chain. The focus groups provide an additional layer of validation by complementing individual interviews with group-based insights, thereby helping to mitigate potential biases or inconsistencies in individual responses.

## 3. Results

### 3.1. Jiaogulan value chain map

**Fig 1:**
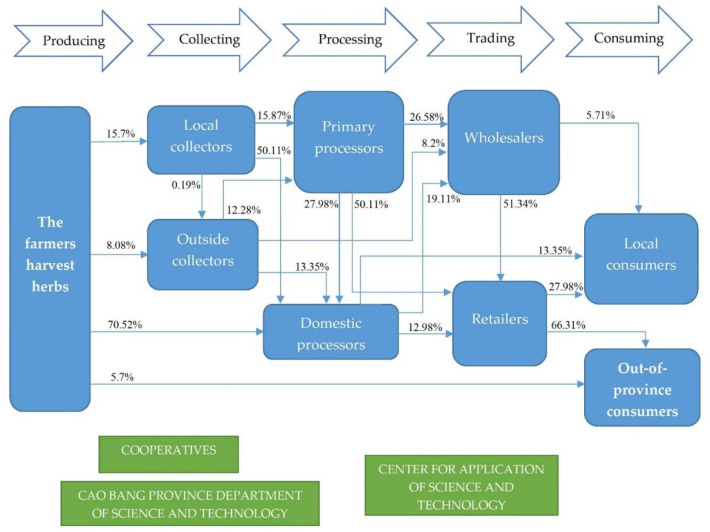
A map of the Jiaogulan value chain in CBGP, Vietnam.

Fig 1 shows that the determined actors involved in the Jiaogulan value chain map in CBGP include farmers, local collectors, outside collectors, preliminary processors, domestic processors, wholesalers, retailers, and local consumers. Farmers who harvest the Jiaogulan herbs, contributing in the initial stage of the value chain. Local collectors gather harvested herbs from farmers and prepare them for preliminary processing. Outside collectors come from neighboring regions to source Jiaogulan, thereby broadening the market reach. Preliminary processors perform initial processing activities, such as drying and sorting the herbs. Domestic processors further refine the product, creating various forms such as teas, extracts, and supplements. Wholesalers purchase processed products in bulk to distribute to retailers. Retailers sell the final products directly to consumers, both in physical stores and online. Local consumers purchase Jiaogulan for personal use. Consumers living outside CBGP access these products through online platforms or other distribution channels.

While some actors in the Jiaogulan value chain are directly involved in the local processes of harvesting, processing, and distribution, there are also individuals from neighboring provinces supporting these activities. These individuals provide essential inputs such as agricultural tools, seeds, and fertilizers, as well as financial resources to assist farmers and processors. Additionally, they offer technical assistance, sharing knowledge about practices in cultivation and processing, which enhances the overall efficiency and quality of the value chain. Their involvement also includes transportation and distribution services, ensuring that Jiaogulan products reach consumers in both local and wider markets. Unlike many agricultural value chains in Vietnam, particularly those in other areas of the Northern mountainous region, the Jiaogulan value chain in CBGP is notably free from the involvement of Chinese or other foreign actors. This absence of external actors is significant, as it allows local communities to maintain control over their resources and production processes. The local-centric structure of the value chain ensures that the economic benefits, such as profits from sales and job creation, remain within the community. This focus on local involvement not only fosters economic development but also promotes sustainability by encouraging the use of local resources, traditional practices, and community engagement, ultimately contributing to the long-term viability of the Jiaogulan industry in the region (Fig 1).

The results of interviews conducted with district and provincial officials, as well as local residents in CBGP, indicate that all actors involved in the Jiaogulan value chain play equally important roles in maintaining a stable structure within the value chain. Each participant, from farmers and collectors to processors and distributors, contributes to the overall functionality and resilience of the market. The Jiaogulan value chain extends beyond CBGP to encompass the entire Cao Bang province, several neighboring northern mountainous provinces such as Bac Kan and Lang Son, and even reaches consumers in Hanoi city.

Several critical factors significantly impact the efficiency and effectiveness of the Jiaogulan value chain. The local climate determines the growth conditions for Jiaogulan, while the state of transport infrastructure is essential for the timely collection and distribution of the herb. Accessibility is another important factor, as many harvesters live in remote moutainous areas with limited access to socio-economic services that could support their activities in collection, transportation, and delivery to consumers.

Harvesting of Jiaogulan primarily occurs in the wild, with cultivated products accounting for only a small proportion of the total supply. The herb is mainly collected by ethnic minorities, typically individuals aged 45–50 years. The collection occurs on sloping land, often under challenging conditions due to the rudimentary transport infrastructure available in these regions. Weather variability, particularly rainfall, can significantly affect harvest yields, making the timing and success of the collection process unpredictable.

Once harvested, Jiaogulan is dried by preliminary processors before being sold to wholesalers operating in the northern mountainous provinces of Vietnam. Although various market channels exist for distributing Jiaogulan, the majority of the herb is funneled through four primary market channels, which transport large volumes of products and generate significant added value throughout the entire chain. Among these, the second channel is responsible for delivering the highest proportion of total Jiaogulan production directly to the local Cao Bang province, while the fourth channel focuses on supplying products to the provincial market. The other channels are characterized as intermediate, catering to limited volumes of the herb, but they still play a role in the overall market dynamics.

* *Channel 1: Famers harvesting herb → retailers → local consumers*

This channel serves as the fastest delivery route from farmers to consumers, ensuring that Jiaogulan reaches the market quickly and efficiently. In this process, Jiaogulan is harvested directly from the wild by farmers who possess deep knowledge of the local environment and optimal harvesting techniques. Once collected, the fresh Jiaogulan is promptly picked up by retailers who specialize in local markets. These retailers connect farmers with consumers, facilitating a direct relationship that benefits both parties.

The products delivered through this channel are primarily fresh Jiaogulan. Households in CBGP utilize fresh herbs directly in their meals, often incorporating them into traditional dishes, herbal teas, or health remedies. This direct consumption underscores the cultural significance of Jiaogulan within local communities, where it is appreciated not only for its health benefits but also as an integral part of local culinary practices. By bypassing intermediaries, this channel speeds up the delivery process and helps retain a greater share of the product’s value within the local economy.

Consumers benefit from accessing fresh and high-quality Jiaogulan, while farmers receive immediate compensation for their harvest, fostering a sustainable and mutually beneficial relationship. The efficiency of this channel highlights the importance of local networks in the Jiaogulan value chain, contributing to food security and economic resilience in the region.

* *Channel 2: Famers harvesting herb → processors → retailers → local consumers*

After harvesting, Jiaogulan undergoes preliminary processing using manual-drying methods at the household level. This method involves carefully spreading the freshly harvested leaves in a well-ventilated area to ensure proper drying without compromising the herb’s quality and nutritional value. The manual-drying process is labor-intensive but allows farmers and local processors to maintain control over the quality of their product, which is crucial for preserving the herb’s beneficial properties.

Once the drying process is complete, the dried Jiaogulan is sold directly to processing facilities within the province. A significant portion of this output, accounting for approximately 70.52%, is supplied to facilities associated with the Department of Science and Technology of Cao Bang province. These processing facilities are equipped to handle larger volumes and employ more advanced techniques to refine the herb, producing various value-added products such as teas, extracts, and dietary supplements. Their involvement not only enhances the marketability of Jiaogulan but also supports the local economy by providing jobs and contributing to technological advancements in processing methods.

In addition to these processing facilities, about 12.98% of the total output from the Jiaogulan value chain is sold directly to retailers in Cao Bang. These retailers, which include vendors operating in local markets, grocery stores, and small shops in both commune and district markets, connect local producers with consumers. By facilitating direct sales, they ensure that fresh and processed Jiaogulan products are readily available to consumers, who increasingly seek natural and health-promoting ingredients for their diets.

* *Channel 3: Famers harvesting herb → outside collectors → wholesalers → retailers → outside consumers*

In this channel, approximately 8.08% of the total volume of Jiaogulan is sold to collectors from other provinces. After harvesting, farmers sell their freshly collected Jiaogulan to these outside collectors, who source the herb from various local producers. These collectors have established networks and knowledge of market demand, allowing them to efficiently aggregate larger quantities of Jiaogulan from multiple farmers.

Once the Jiaogulan is collected, it is sold to wholesalers who are typically based in other provinces. These wholesalers are vital intermediaries in the value chain, as they purchase the herb in bulk and ensure that it meets the quality standards required for further distribution. They operate on a larger scale, consolidating the product and negotiating better prices, ultimately facilitating wider market access.

Transportation is a significant aspect of this channel. The wholesalers utilize large-tonnage trucks to transport the collected Jiaogulan to various destinations, often crossing provincial borders. This logistics network is essential for distributing the herb efficiently, ensuring that it reaches both urban and rural markets outside Cao Bang province.

Upon reaching their destinations, the wholesalers distribute the Jiaogulan to a network of retailers. The end consumers in this channel are typically located in different provinces or even in urban centers, where there is a growing demand for natural health products and herbal remedies.

This channel highlights the broader market reach of Jiaogulan beyond its local origins, showcasing how it can be distributed to consumers seeking its health benefits. By connecting local farmers with outside collectors and ultimately to consumers in other provinces, this channel expands the market for Jiaogulan and supports the livelihoods of those involved in its production and distribution. This dynamic enhances economic opportunities for farmers and collectors and promotes the cultural significance of Jiaogulan as a valued herbal product in various regions.

* *Channel 4: Famers harvesting herb → local collectors → processing facilities → wholesalers → retailers → outside consumers*

In this channel, farmers harvest Jiaogulan and sell it to both local collectors and outside collectors. These collectors aggregate the herb from various farmers, ensuring that a sufficient volume is available for processing. They maintain relationships with farmers, help negotiate prices, and facilitate the efficient movement of fresh products to processing facilities. Approximately 50.11% of the total Jiaogulan products in this value chain are purchased by processors located outside the province. These processing facilities are equipped to handle larger quantities of raw Jiaogulan, employing methods to transform the fresh herb into a variety of final products. Pre-processed Jiaogulan undergoes several steps to enhance its marketability and shelf life. For instance, the raw herb may be dried, cut, or blended to create products such as tea bags and dried tea leaves. These products are then packaged for distribution, ensuring they retain their flavor, aroma, and health benefits.

Once processed, the finished Jiaogulan products are sold to wholesalers who specialize in distributing herbal goods. These wholesalers consolidate the processed products and transport them to various retailers. This network includes health food stores, supermarkets, and online marketplaces, where the final products become accessible to a wider audience. Retailers then market these Jiaogulan products to consumers, many of whom are located outside the province. This channel accounts for approximately 27.98% of the total volume of Jiaogulan sold to final consumers. These consumers are often health-conscious individuals seeking natural remedies and herbal supplements to enhance their well-being. The availability of Jiaogulan in diverse retail settings allows for greater exposure and accessibility, tapping into the growing demand for herbal products in both urban and suburban markets.

### 3.2. Value added of actors in market channels of value chain

Harvesters/farmers participate in all market channels of the Jiaogulan value chain. In each channel, the cost of farmers has not changed, the main difference among market channels is the added value per ton of Jiaogulan. Table 2 shows the information on added value and net added value.

**Table 2:** Added value and net added value of famers in the main channel of Jiaogulan value chain (unit: $USD per ton).

Items	Famers	Collectors	Wholesalers	Retailers
*Channel 1: Famers harvesting herb → retailers → local consumers*				
Price (USD)	0.209			0.399
Intermediary costs	0.029			0.300
Added value	0.180			0.098
Added cost	0.070			0.069
Net added value	0.110			0.029
Percentage of net added value	79.060			20.940
*Channel 2: Famers harvesting herb → processors → retailers → local consumers*				
Price	0.209	0.285		0.394
Intermediary costs	0.029	0.209		0.285
Added value	0.181	0.076		0.109
Added cost	0.070	0.023		0.016
Net added value	0.110	0.052		0.094
Percentage of net added value	43.030	20.390		36.580
*Channel 3: Famers harvesting herb → outside collectors → wholesalers → retailers → outside consumers*				
Price	0.209	0.297	0.409	0.496
Intermediary costs	0.029	0.209	0.297	0.409
Added value	0.181	0.087	0.113	0.087
Added cost	0.070	0.023	0.027	0.016
Net added value	0.110	0.064	0.085	0.072
Percentage of net added value	33.280	19.310	25.800	21.610
*Channel 4: Famers harvesting herb → local collectors → processing facilities → wholesalers → retailers → outside consumers*				
Price	0.207	0.258	0.409	0.496
Intermediary costs	0.029	0.207	0.296	0.409
Added value	0.178	0.051	0.113	0.087
Added cost	0.070	0.015	0.027	0.016
Net added value	0.108	0.036	0.086	0.072
Percentage of net added value	33.660	11.190	26.840	22.410

Table 2 presents a comprehensive analysis of the four channels through which Jiaogulan is marketed, highlighting significant differences in added value across these pathways. Channel 2 and channel 4 exhibit the highest levels of added value, indicating more profitable avenues for farmers.

In channel two, farmers sell their harvested Jiaogulan directly to processing facilities at a price of $0.209 USD per ton. This transaction generates an added value of $0.18 USD per ton for the farmers. After accounting for intermediary costs and additional expenses, the net added value realized by the farmers amounts to $0.11 USD per ton. The advantage of this channel is that processors at CBGP purchase fresh Jiaogulan directly from farmers, bypassing local collectors. This direct transaction model helps minimize added costs, ultimately benefiting the farmers. Local collectors possess a keen understanding of the psychological factors that influence local harvesters, allowing them to negotiate purchase prices that are typically lower than those offered by external collectors and processing facilities. This dynamic reflects the local collectors’ awareness of the market conditions and relationships, which can sometimes place farmers at a disadvantage.

In channel 4, the situation is slightly different. Farmers sell Jiaogulan to local collectors at a price of $0.207 USD per ton, which is notably lower than the selling price to processing facilities, recorded at $2.683 USD per ton. When farmers opt to sell directly to retailers, they achieve a selling price of $0.208 USD per ton. The added value generated by farmers in this channel remains at $0.18 USD per ton, while their net added value is slightly lower at $0.109 USD per ton.

When examining the distribution of added value across all four market channels, it becomes evident that farmers receive a significant proportion of the added value, with percentages ranging from 33.28% to 79.6%. This statistic underscores the relatively favorable position of farmers within the value chain. Although the differences in added value and net added value among the various market channels are not statistically significant, channel 2 stands out as the most lucrative for farmers. By selling directly to processing facilities, farmers can secure a higher income, thereby reinforcing the notion that developing an efficient market channel for processed Jiaogulan could substantially enhance farmers’ earnings.

Overall, the findings illustrate that a direct distribution channel, where farmers sell directly to processing enterprises, proves to be more profitable, ultimately contributing to an increase in income for the farmers involved. This insight suggests that strategic improvements in market access and infrastructure could further amplify the benefits for local agricultural producers.

### 3.3. Impact of added value and significance of Jiaogulan products on household income

Table 3 provides a quantitative analysis of the relationship between added value and farmers’ income in the context of Jiaogulan cultivation. The results indicate that even a modest increase in added value can have a measurable impact on the economic well-being of farmers. For instance, if added value doubles, farmers can expect their income to rise by approximately $3.10 USD per hectare. This incremental gain reflects the direct benefits that can be realized from enhancing the value of their agricultural products and highlights the potential for farmers to improve their livelihoods through strategic enhancements to the value chain.

**Table 3:** Impact of value-added and distribution of Jiaogulan products on the income of farmers.

Impacts	Value added (USD)	Increase 10%	Increase 15%	Increase 20%	Increase 100%
Impacts of added value and net added value	Income (USD/hectare)	0.310	0.464	0.619	3.100
	Income from Jiaogulan (USD/household)	0.281	0.422	0.562	2.810
	Income (USD/hectare)	0.189	0.283	0.377	1.890
	Income from Jiaogulan (USD/household)	0.171	0.257	0.343	1.710
Impacts of distribution added value and net added value	Income (USD/hectare)	0.482	0.723	0.964	4.820
	Income from Jiaogulan (USD/household)	0.452	0.678	0.904	4.520
	Income (USD/hectare)	0.539	0.809	1.079	5.390
	Income from Jiaogulan (USD/household)	0.506	0.759	1.012	5.060

When assessing income on a household level across the total harvested area of Jiaogulan, similar patterns emerge. A doubling of added value translates to an average income boost of $2.81 USD per household. This finding underscores the importance of added value in uplifting household incomes, particularly in regions where farmers rely heavily on their agricultural outputs.

In addition to added value, the impact of net added value on farmers’ income is also noteworthy. If net added value doubles, farmers’ income is projected to rise by $1.89 USD per hectare and $1.71 USD per household. Given that many individuals living in the CBGP belong to impoverished ethnic minority groups, these increases in both added value and net added value are crucial indicators of improving local economic conditions. The Jiaogulan product value chain enhances the income levels of these communities, contributing to poverty alleviation and the sustainability of the local economy.

Further analysis reveals that an increase double in added value leads to an income increase of $4.82 USD per hectare. In scenarios where the rate of added value distribution increases by 20%, the income rise is even more pronounced, reaching $0.964 USD per hectare. On a household basis, a doubling of added value results in a $4.52 USD increase in income per household. If the distribution of added value were to improve by doubling, farmers would see an increase in income of $5.39 USD per hectare. The findings illustrate that even a slight increase in added value can lead to significant improvements in household income.

As farmers generate added value through their cultivation practices, the equitable distribution of this added value becomes essential. Enhanced distribution mechanisms increase income levels for farmers. These insights underline the importance of developing and optimizing the Jiaogulan value chain to maximize economic benefits for local farmers and contribute to the broader goal of sustainable development in the region.

## 4. Discussions

The study findings address issues related to strengthening value chain links for Jiaogulan, a key NTFP for smallholder farmers in CBGP, Vietnam, by using the VCA approach. In developing countries, NTFPs and other agricultural value chains are characterized by complex linkages and governance structures, including both horizontal and vertical connections among actors [37–40]. Horizontal links typically involve collaboration among similar actors, such as small-scale farmers within cooperatives [41,42], while vertical links are formalized through contracts managed by companies or organizations, ensuring transparent commodity flows and quality control [43,44]. Developing vertical linkages can effectively upgrade value chains and reduce poverty by lowering production costs, increasing output, and more equitably distributing benefits [45]. In this study, the links in the Jiaogulan value chain at CBGP reflect typical characteristics of value chains involving smallholder farmers. Study results indicate that these links are relatively weak, with loose production cooperation, a lack of leading actors, and an absence of a transparent market information system. The main reasons for these issues include limited production capacity, underdeveloped trademarks, and inadequate market access for producers. Cooperative economic organizations struggle to organize and enhance production capacity effectively, and production planning remains incomplete and fragmented. Also, the information infrastructure is underdeveloped, and the application of digital technology in product marketing is minimal to non-existent.

In the Jiaogulan value chain in BCGP, local farmers generate the highest added value, followed by wholesalers and processors, indicating that direct participation in production stages significantly increases income. This finding is consistent with studies showing that farmers add more value by engaging directly in production [46–48]. For example, in Nigeria, women farmers who process cassava create higher added value than those selling fresh cassava [40]. Similarly, farmers in the banana value chain generate the highest profits, highlighting their critical role despite a lower share of value-added distribution [49]. However, contrasting results have been observed. For instance, in the pouring value chain in Wonogiri Regency, processors capture 97.88% of added value, while farmers receive a minimal share [50]. In the Brazilian aquaculture value chain, small-scale fish farmers are often excluded from processing industries, resulting in low added value [51]. The distribution of net added value varies among actors depending on the market channel, with farmers generally gaining the highest net value in the main channels. This distribution is influenced by marketing efficiency, value chain governance, and the level of cooperation among actors [52,53].

Uneven value addition can diminish benefits to farmers if they are excluded from direct distribution channels, a common issue where intermediaries take a larger share, leaving farmers with minimal profits [54]. Bypassing intermediaries and selling directly can increase farmers’ benefits and satisfaction [55]. Conversely, in Turkey’s groundnut value chain, downstream actors hold strong positions against farmers due to neoliberal policies, which have increased production costs without corresponding income growth [56]. Eliminating intermediaries could enhance small farmers’ positions by reducing barriers to market entry.

The dynamics among actors in the chain highlight the need for cooperation between enterprises and small farmers to improve farmers’ positions, increase income, and strengthen the agricultural value chain [49,57]. This study extends the understanding of the Jiaogulan value chain by quantifying the added and net value farmers receive in different distribution channels. It suggests that direct distribution channels could optimize farmers’ benefits, increasing their income by 20–25% compared to those using intermediary channels. These findings provide a basis for policy recommendations to promote direct distribution and strengthen connections among cooperative partners in the chain.

The added value and net added value from Jiaogulan collection in CBGP significantly contribute to farmers’ total income. However, despite their contribution, farmers are the least efficient actors in terms of investment efficiency, earning lower incomes compared to traders. Studies by Dimitrijević and Despotović (2023) [58] indicate that when farmers focus on product quality and sell directly to international markets, their incomes increase significantly. For example, integrating smallholders into the pineapple export chain has bolstered rural economic growth by creating employment opportunities and strengthening local producer-consumer networks, ultimately improving rural welfare and income [59]. This approach offers a potential solution for Jiaogulan farmers in CBGP, where international markets are still underutilized. Promoting product quality and establishing access to international markets can help increase farmers’ incomes and reduce their reliance on domestic intermediaries. Therefore, policies aimed at enhancing the benefits for Jiaogulan collectors are necessary.

This study have some limitations. While the study examines the role of Jiaogulan production and trade in household income generation, it does not provide a detailed comparative analysis of its significance relative to other income sources or its direct impact on poverty alleviation. This is a limitation of the study, as a more comprehensive household income assessment, including poverty thresholds, would have strengthened the findings. Future research could address this gap by incorporating household expenditure surveys and poverty impact assessments to determine the extent to which Jiaogulan contributes to economic resilience. Similarly, while the study identifies disparities in economic benefits among stakeholders in the value chain, it does not fully explore the underlying factors driving these inequities, such as gender, age, or ethnicity. We recognize that these social dimensions may influence access to resources, market opportunities, and overall benefit distribution. This represents another limitation of the study, and future research should integrate a more intersectional approach, using disaggregated data and qualitative insights to better understand the socio-economic barriers affecting different groups within the Jiaogulan value chain.

The Central Government of Vietnam has implemented various policies to promote sustainable agricultural development, focusing on high-value products, value chain development, and the prioritization of organic agriculture. These policies are designed to increase the added value of agricultural products, and to manage market risks. Recent national agricultural policies have been issued and implemented including those encouraging cooperation and linkages between production and consumption of agricultural products (2018); renewing agri-business activities for 2021–2025 with a long-term outlook to 2030 (2021); restructuring Vietnam’s agricultural sector for 2021–2025 (2021); and establishing sustainable development policies for agriculture and rural sectors for 2021–2030, with a vision to 2050 (2022). The central government of Vietnam has targeted the development of medicinal plant value chains to shift production toward market-oriented practices, prioritize domestic pharmaceutical products, and leverage high-value endemic medicinal plants for both domestic and international markets. Key policies include the Law on Forestry (2017); a master plan for herbal medicinal plant development up to 2020, with an outlook to 2030 (2013); plans for the pharmaceutical and herbal medicine industry development by 2030, with a vision to 2045 (2021); and Vietnam’s forestry development strategy for 2021–2030, with a vision to 2050 (2021).

In CBGP, agriculture is a crucial economic sector, with rare and valuable medicinal plants serving as key natural resources. The local government has implemented policies including those aimed at enhancing linkages in agricultural production and consumption (2018); encouraging investment from enterprises and cooperatives in agriculture and rural areas (2019); promoting the development of smart agriculture for 2020–2025, with a vision to 2030 (2019); and advancing agriculture and forestry (2020). Based on the research findings, the following policy recommendations are proposed to enhance collaboration among stakeholders in the Jiaogulan value chain: strengthening production resources by investing in technical training for farmers in Jiaogulan cultivation, processing, and distribution; improving productivity and product quality; and integrating Jiaogulan production into the master plan of Cao Bang Province up to 2030, with a vision to 2050. Developing eco-labels for Jiaogulan products and expanding market access to segments such as supermarkets and safe food stores are essential. Cooperation should be enhanced by strengthening vertical and horizontal links among stakeholders through legal contracts that ensure mutual benefits. Distribution channels for producers should be diversified and developed sustainably, with a focus on promoting transparency in product quality. Retailers must provide accurate information on product quality and sourcing, while collectors and wholesalers should formalize transactions through binding commercial contracts to enhance quality control and pricing.

Local governments should invest in agricultural infrastructure, including irrigation, electricity, communication, transportation, warehouses, and markets, to better connect Cao Bang with other regions. Policies should facilitate access to credit and loans for agricultural technology and herbal medicinal value chains. Additionally, local governments should implement policies to establish trademarks for Jiaogulan products, such as geographical indications and eco-labels, and support producers and consumers in accessing market information. A digital information system for e-commerce should be developed, providing market news and production applications available on mobile devices. Policies should also support the establishment of local agricultural trading e-commerce platforms.

While e-commerce and international standards offer market expansion opportunities, small-scale Jiaogulan producers face significant barriers to implementation. Limited digital literacy, internet access, and financial resources hinder their ability to adopt e-commerce platforms and manage online sales. The costs of digital marketing, logistics, and payment systems further add to the challenge. Compliance with international standards (e.g., organic certification, GAP) requires costly upgrades in production, documentation, and testing. Certification fees, quality control measures, and structural adjustments in farming and processing impose financial burdens that many small producers cannot afford. To overcome these challenges, targeted policy support such as subsidies for certification, training in digital marketing, and investment in rural e-commerce logistics, would be essential. Encouraging cooperatives to share costs and resources could also improve small-scale producers’ competitiveness in domestic and international markets.

To promote the medicinal product value chain, the provincial government should implement policies that encourage cooperation between the production and distribution of medicinal products, including supportive land, tax, and credit policies. These policies should promote local participation in the conservation and development of medicinal plants, including Jiaogulan, and support the cultivation of medicinal products according to good agricultural practices while protecting producers’ rights.

Since local Jiaogulan products are primarily consumed domestically, expanding into international markets is essential. Policies should support training and facilitate access to technology and international standards, such as the Global Good Agricultural Practice (GlobalGAP), to enhance product quality and market penetration.

Moreover, the Vietnam government should concern about the long-term sustainability of the Jiaogulan value chain, which depends on responsible cultivation and harvesting practices. Overexploitation, particularly in wild harvesting, could lead to resource depletion and biodiversity loss. Small-scale farmers may also face challenges in maintaining soil health and sustainable yields due to limited knowledge of agroecological practices. Encouraging GlobalGAP, crop rotation, and reforestation efforts can help mitigate these risks and ensure long-term viability. Beyond cultivation, the environmental impacts of processing, packaging, and distribution remain a concern. The use of chemicals in preliminary processing, plastic packaging materials, and carbon emissions from transportation contribute to ecological footprints. Implementing eco-friendly packaging, energy-efficient processing technologies, and sustainable logistics strategies is crucial for reducing the environmental impact of the value chain. To enhance sustainability, policy incentives, producer education, and green certification programs should be promoted, encouraging environmentally responsible practices throughout the Jiaogulan industry.

While this study focuses on the Jiaogulan value chain in Cao Bang Geopark, its findings and recommendations have broader relevance to other regions with similar non-timber forest products (NTFPs). Many challenges identified such as market access, sustainability concerns, and policy support, are shared by other NTFPs in rural economies. However, variations in ecological conditions, regulatory frameworks, and local socio-economic contexts may influence the applicability of interventions. Future research should explore comparative analyses across regions to refine context-specific strategies. Additionally, the study does not explicitly outline mechanisms for monitoring and evaluating the impact of proposed interventions, which is critical for assessing their effectiveness. To ensure sustainable development, key performance indicators (KPIs) such as income growth, market expansion, environmental impact, and stakeholder participation, should be tracked over time. Implementing regular impact assessments, stakeholder feedback mechanisms, and pilot programs would help refine policies and adapt strategies to evolving market and environmental conditions.

## 5. Conclusions

The Jiaogulan value chain in CBGP, Vietnam, is characterized by a diverse array of actors, including farmers, local and outside collectors, processors, wholesalers, and retailers. This structure facilitates the collection, processing, and distribution of Jiaogulan, enabling the herb to reach both local and broader markets, including urban consumers in Hanoi city capital of Vietnam. Local collectors and outside collectors aggregate Jiaogulan from farmers, providing essential inputs, technical assistance, and market connections. This involvement enhances the efficiency and quality of the value chain while promoting local control over resources and production processes. The value chain is devoid of overseas actors, such as those from China, which allows local communities to retain economic benefits and ensures that profits and job opportunities remain within the region. This local-centric model supports sustainability by leveraging local resources and traditional practices.

Four primary market channels facilitate the distribution of Jiaogulan, each contributing differently to added value. Channels that connect farmers directly to processing facilities and those involving local collectors and processors demonstrate higher added value for farmers. The findings illustrate a direct relationship between added value and household income for farmers. Even modest increases in added value can lead to substantial income improvements, highlighting the importance of optimizing the value chain for poverty alleviation and economic development.

Overall, the Jiaogulan value chain studied in CBGP presents a significant opportunity for local economic development, with the potential to improve the livelihoods of farmers and promote sustainable practices within the community. Strategic interventions aimed at enhancing value addition and market access are essential for realizing these benefits.

## Supporting information

S1 File Data English version. (XLSX)
